# Statistical analysis of annual maximum daily rainfall for Nelspruit and its environs

**DOI:** 10.4102/jamba.v10i1.499

**Published:** 2018-03-26

**Authors:** Eric M. Masereka, George M. Ochieng, Jacques Snyman

**Affiliations:** 1 Department of Civil Engineering, Tshwane University of Technology, South Africa; 2 Department of Civil Engineering, Vaal University of Technology, South Africa

## Abstract

Nelspruit and its environs frequently experience extreme high annual maximum daily rainfall (AMDR) events resulting in flood hazards. These flood hazards have caused flood disasters that have resulted in loss of property and lives. The main objective of this study was to carry out statistical analysis of extreme high AMDR events that have caused flood hazards, which in turn have caused flood disasters in Nelspruit and its environs. Empirical continuous probability distribution functions (ECPDF) and theoretical continuous probability distribution functions (TCPDF) were applied to carry out the statistical analysis of the extreme high AMDR events. Annual maximum daily rainfall event of magnitude 100 mm was identified as a threshold. Events > 100 mm were considered as extreme high events resulting in flood disasters. The results of empirical frequency analysis showed that the return period of flood disasters was 10 years. The occurrence probability of flood disaster event at least once in 1, 2, 3, 4 and 5 years was 0.10, 0.19, 0.27, 0.34 and 0.41, respectively. Generalised logistic PDF was identified as the best-fit theoretical PDF for statistical analysis of the extreme high AMDR events in Nelspruit and its environs. The results of this study contributed to the understanding of frequency and magnitude of extreme high AMDR events that could lead to flood disasters. The results could be applied in developing flood disaster management strategies in Nelspruit and its environs.

## Introduction

Extreme high annual maximum daily rainfall (AMDR) events are among environmental events that have caused the most disastrous consequences for human society (Kysely, Picek & Huth 2006). Flood hazards caused by high extreme rainfall events have resulted in flood disasters that have accounted for 47% of all weather-related disasters affecting 2.3 billion people worldwide (Wahlstrom & Guha-Sair 2015). Death tolls because of floods have also risen in many parts of the world. In 2007, floods killed 3300 people in India and Bangladesh alone; in 2010, floods killed 2100 people in Pakistan and 1900 people in China, whereas in 2013, 6500 people died because of floods in India (Wahlstrom & Guha-Sair 2015). These flood events have been attributed to effects of human-induced climate change (Komi, Amisigo & Diekkriiger 2016). In order to formulate and develop strategies to manage and reduce flood disaster risk, it is necessary to carry out statistical analysis of the AMDR events that cause these flood disasters.

Occurrences of floods in Nelspruit and its environs because of extreme high AMDR events have been documented. On 29 January 1974, heavy rains of 112.6 mm in 24 h caused floods that destroyed property in Nelspruit and its environs (SAWS 1994). On 29 January 1984, heavy rains of 110.5 mm in 24 h caused floods in which four lives were lost and several agricultural dams were destroyed (Kovacs et al 1984). On 08 February 1985, 126.0 mm of rain in 24 h caused damage to roads and bridges in Nelspruit and its environs (SAWS 1985). The heavy rains and storms of 106.2 mm on 06 February 2000 followed by 102.8 mm on 07 February 2000 caused a lot of damage. It was estimated that 1240 km of paved roads, 1306 km of gravel roads, 120 km of farm roads and 84 bridges were damaged. Also, these floods destroyed 16 large dams and 96 small farm dams (Smithers et al. 2000). On 18 January 2012, heavy rains of 109.0 mm caused floods and extensive damage in Nelspruit and its environs (News24 2012). On 11 March 2014, heavy rains of 107.3 mm in 24 h which was followed by 104.8 mm of rain of the previous 24 h caused the death of 11 people in Nelspruit and its environs (News24 2014).

The areas that have been affected by flood hazards are mainly in the river courses and more so in the flood zones. Bridges, water pumping stations, farm roads and irrigation systems are among the infrastructures that are frequently affected by flood hazards.

Despite the frequent extreme high AMDR events that have been causing floods resulting in loss of human lives and property, limited research has been carried out on the frequency and magnitude of extreme high AMDR events in Nelspruit and its environs (SAWS 2014).

Approaches to reduce flood disaster risk caused by extreme high AMDR include flood disaster mitigation, early warning systems, disaster preparedness, recovery and support livelihood (Komi et al. 2016). Of these approaches, flood mitigation plays a pivotal role. Mitigation of flood disaster can be carried out in two methods, namely engineering method to control floods and regulatory method designed to decrease flood vulnerability (Komi et al. 2016). The engineering method includes construction of structures like channel modifications, retention walls, levees and dikes. The regulatory method includes flood plain zoning and building construction codes. Results of statistical analysis of high extreme AMDR events are necessary to develop and formulate methods and strategies for flood disaster reduction and mitigation.

The aim of this study was to carry out statistical analysis of extreme high AMDR events which cause flood hazards that result in flood disasters in Nelspruit and its environs. The other objective of the study was the identification of the theoretical probability distribution function(s) (PDF) that best describe these extreme high AMDR events which cause flood hazards that result in flood disasters in Nelspruit and its environs.

The initial studies of rainfall events that caused flood disasters in Nelspruit and environs were concentrated on atmospheric mechanisms that resulted in these heavy rainfall events. Simpson (1996) studied cumulus clouds and the associated larger mesoscale systems that produced heavy storms in and around Nelspruit Schulze (1972) from his studies concluded that there was a high incidence of hail and thunder in North Eastern region of South Africa. Kelbe (1984) studied cumulus cloud characteristics and observed that most of the severe storms occurred in the early summer months in the radius of 50 km around Nelspruit. Studies on variability and probability of rainfall in relation to coefficients of variation of monthly and annual rainfall series at Nelspruit have been carried out. Green (1969) came to a conclusion that the Type 111 model, which had two independent parameters q and p, was widely applicable in describing probability of monthly rainfall series. Recently, Mackellar, New and Jack (2014) in the study of observed and modelled trends in rainfall and temperature for South Africa for the period of 1960–2010 reported that a cluster of rainfall stations in the Lowveld in Mpumalanga showed increase in precipitation. None of the cited studies were focused on the frequency and magnitude analysis of extreme high AMDR events which caused flood hazards that resulted in flood disasters in Nelspruit and its environs. This study was therefore carried out to fill the gap of frequency and magnitude analysis of AMDR events which cause flood hazards that result in flood disasters in Nelspruit and its environs which was the focus of this study.

The steps of statistical analysis of hydrometeorological events involve selecting PDF to describe the phenomenon of interest, estimating parameters of that function and thus obtaining the risk estimates of satisfactory accuracy for the problem at hand (Stedinger, Vogel & Batchelder 2001). Several PDFs have been selected and applied for frequency analysis of rainfall events. In United States of America (USA), Naghavi and Yu (1995) applied generalised extreme value (GEV) distribution function and found it suitable for frequency and magnitude analysis of AMDR events in Louisiana State. Daud et al. (2002) identified GEV as the best-fit PDF for frequency analysis of AMDR events in Malaysia. Park et al. (2010) also identified GEV as the best-fit PDF for frequency analysis of AMDR events in South Korea. However, other PDFs have also been identified as best-fit PDFs for frequency analysis of AMDR events. Olumide, Saidu and Oluwasesan (2013) in the study of frequency analysis of rainfall events at Tagwai Dam in Nigeria identified normal and Log-Gumbel PDFs as the best-fit PDFs for frequency analysis of the AMDR events. Goula Bi et al. (2010) identified and applied Gumbel and log normal PDFs for frequency analysis of AMDR events at 43 rainfall stations in Cot de Ivoire. Mason et al. (1999) identified Beta-K and Beta-P distributions as the best-fit PDFs for frequency analysis of AMDR events in South Africa. Du Plessis and Burger (2015) identified GEV as the best-fit distribution function to analyse the frequency of short-duration rainfall intensities in Western Cape Province in South Africa. In the study of cumulus cloud characteristics of the Eastern Transvaal Lowveld, rainfall events of 100 mm or more in 24 h were identified as the events that resulted in floods in Nelspruit and its environs (Kelbe 1984). In this study, AMDR event of magnitude of 100 mm was adopted as a threshold. AMDR events of greater than 100 mm were considered as events which cause flood hazards resulting in flood disasters. Also in this study, a method based on ranking of statistics of chi-squared (CS), Kolmogorov–Smirnov (KS) and Anderson–Darling (*A*^2^) goodness-of-fit tests developed by Masereka et al. (2015) were applied to selected candidate and best-fit PDF for the analysis of magnitude and frequency of the AMDR events in Nelspruit and its environs.

## Materials and methods

### Location and climate

**FIGURE 1:**
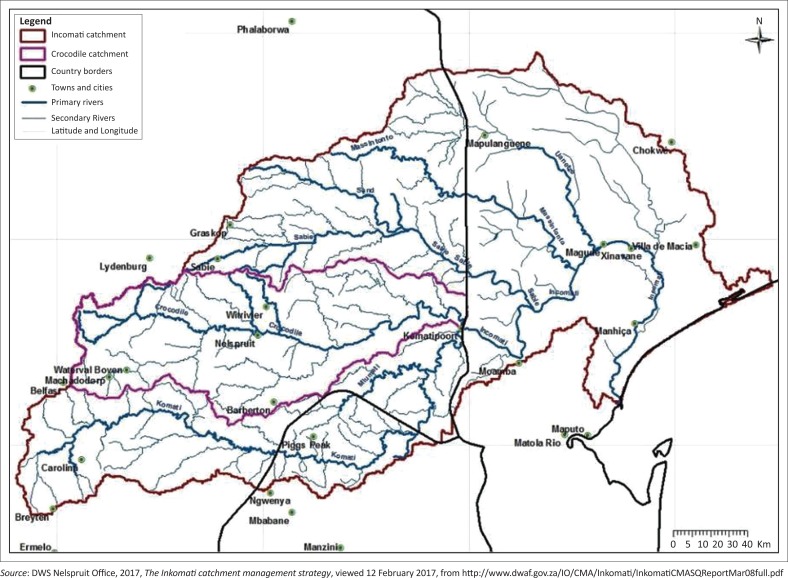
Drainage system of Incomati catchment showing the position of Nelspruit and its environs.

**FIGURE 2:**
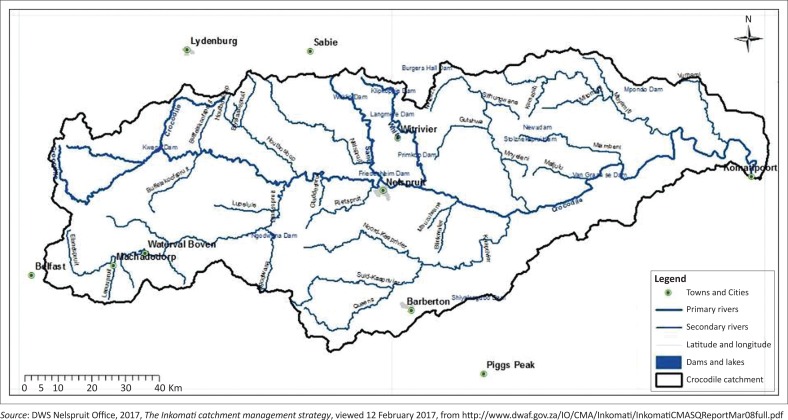
The Crocodile River catchment showing Nelspruit and its environs.

Nelspruit is located 330 km east of Pretoria in Crocodile River catchment (Figure 1). Crocodile River catchment is part of Incomati catchment (Figure 2). Its geographical coordinates are 25°27′S and 30°58′E. Its altitude is 667 m a.s.l. Nelspruit normally receives about 667 mm of rain per year with most rainfall occurring in months of December, January, February and March. On average, it receives the lowest rainfall (2 mm) in June and the highest rainfall (119 mm) in December. The monthly distribution of average daily maximum temperatures shows that the average midday temperatures for Nelspruit range from 21.4 °C in June to 27.9 °C in January. The region is the coldest during July when the temperature drops to 6.2 °C on average during the night. Maps of Crocodile River catchment and Incomati catchment are shown in Figures 1 and 2.

### Data

The daily rainfall (mm) data for the years 1961–2015 for Nelspruit were obtained from WR 90 and WR 2005 studies, which were carried out by Water Research Council (WRC) (Middleton & Bailey 2005). In these studies, the methods of ensuring and improving quality of daily rainfall data including data used in this study were outlined (Middleton & Bailey 2005). From the daily rainfall data of each year, the largest annual daily rainfall event was extracted. The extracted largest annual daily rainfall events for the years 1961–2015 formed the AMDR events. The AMDR events for the period 1961–2015 are presented in Table 1.

**TABLE 1:** Annual maximum daily rainfall (mm) events for Nelspruit (1961–2015).

Year	Rainfall (mm)
1961	88.40
1962	36.80
1963	0.00
1964	56.50
1965	56.20
1966	65.10
1967	72.20
1968	57.60
1969	57.00
1970	37.70
1971	67.20
1972	75.60
1973	0.00
1974	112.60
1975	58.00
1976	72.40
1977	40.60
1978	60.50
1979	57.00
1980	77.00
1981	59.40
1982	55.30
1983	78.00
1984	110.50
1985	126.00
1986	49.50
1987	64.70
1988	73.20
1989	43.50
1990	57.70
1991	83.50
1992	48.00
1993	24.50
1994	46.80
1995	47.40
1996	81.40
1997	46.00
1998	56.00
1999	74.80
2000	106.20
2001	34.60
2002	52.20
2003	37.10
2004	44.20
2005	67.00
2006	89.20
2007	64.50
2008	55.60
2009	100.30
2010	57.90
2011	68.80
2012	102.40
2013	79.00
2014	97.30
2015	37.80

## Methodology

### Empirical frequency analysis

Empirical continuous probability distribution function (ECPDF) was applied to determine the return periods of the AMDR events for Nelspruit and its environs for the period 1961–2015. The probability of exceedances (*p*) of the AMDR events was determined by the rank-order method. This method involved ordering the events from the largest event to the smallest event. Rank 1 was assigned to the largest event and rank 55 to the smallest event. The data sample size was 55 events.

To obtain *p* of each event, Weibull formula (Weibull 1939) was applied:

p=i/n+1 [Eqn 1]

*p* is the exceedance probability for an event with rank i, i is the rank of the event, n is the sample size that was equal to 55 in this case. The return period (T) of each event is defined as the inverse of its exceedance probability (Weibull 1939):

T=1/p [Eqn 2]

The empirical return period of each AMDR event was determined by applying equation 2.

### Flood disaster risk analysis

The AMDR events ≥ 100 mm were identified as the events that caused flood hazards resulting in flood disasters in Nelspruit and its environs (Kelbe 1984). AMDR event of magnitude 100 mm was adopted as threshold. AMDR events of magnitude 100 mm or ≥ 100 mm were taken as flood causing events, therefore flood disaster events. Based on this adoption, the model for flood disaster risk analysis (exceedance probability of event *X* being equal to or greater than threshold *x*_*T*_ at least once in *N* years) was developed as demonstrated below.

Letting *x*_*T*_ be the threshold of AMDR variable. The exceedance probability (*p*) of *X* being equal to or greater than *x*_*T*_ at least once in *N* years of record was formulated as:

$$p=P(X\geq x_{T})$$ [Eqn 3]

$$P=p(X<x_{T})=(1-p)$$ [Eqn 4]

$$P(X\geq x_{T}\;at\;least\;once\;in\;N\;years)=1-P(X<x_{T}\;all\;N\;years)$$ [Eqn 5]

$$P(X\geq x_{T}\;at\;least\;once\;in\;N\;years)=1-{\left(1-p\right)}^{N}=1-{\left(1-\frac{1}{T}\right)}^{N}$$ [Eqn 6]

Equation 6 was applied to determine the occurrence probability of flood disaster risk associated with AMDR events of magnitude ≥ 100 mm at least once in 1, 2, 3, 4 and 5 years.

### Stochastic frequency analysis

Theoretical continuous probability distribution functions (TCPDFs) were applied to estimate quantiles of AMDR events of return periods up to 50 years. The estimation of the quantiles was carried out by first identifying the TCPDFs which adequately fitted the AMDR events. Masereka et al. (2015) have developed a methodology to identify candidate and best-fit TCPDFs for frequency analysis of hydrometeorological events. This methodology was adopted to identify candidate and best-fit TCPDFs for frequency analysis of the AMDR events. The identified candidate TCPDFs were subjected to three goodness-of-fit tests, namely CS, Kolmogorov–Smirnov (KS) and Anderson–Darling (AD), to identify the best-fit TCPDF.

### Description of the goodness-of-fit tests

Three goodness-of-fit tests which were applied to identify the best-fit TCPDF from the candidate TCPDFs are described below.

#### Chi-square (*x*^2^) test

Chi-square (*x*^2^) is a goodness-of-fit test that compares how well the TCPDF fits the ECPDF. The chi-square statistic is defined as (Olofintoye, Sule & Salami 2009):

$$x^{2}=\sum_{i=1}^{k}\frac{{\left(O_{i}-E_{i}\right)}^{2}}{E_{i}}$$ [Eqn 7]

where *x*^2^ *is the test statistic, O_l_* is the observed frequency in each category and *E_l_* is the expected (theoretical) frequency in the corresponding category calculated by:

$$E_{l}=F(x_{2})-F(x_{1})$$ [Eqn 8]

where *F* is the cumulative distribution function (CDF) of TCPDF being tested, and *x_1_* and *x_2_* are the lower and upper limits of category i, where *i* runs from 1, … … … *k* and *k* is the number of cells.

#### Kolmogorov–Smirnov test

The Kolmogorov–Smirnov (KS) test is a non-parametric test applied to test whether the sample under consideration is from a reference or hypothesised distribution or to compare whether two samples come from identical distribution (Kottegoda & Rosso 1998). The KS test statistics are calculated from the largest vertical difference in absolute value between the theoretical value and the empirical cumulative distribution functions.

By definition, if a random sample, *x*_1_, *x*_2_ … … … *x_n_*, is from the same distribution with CDF *F*(*x*_1_), then KS test statistic *D = max (D*^+^, *D*^-^*)* where:

$$D^{+}=\max\left(\frac{i}{n}-F(x_{1})\right)$$ [Eqn 9]

and

$$D^{-}=\max\left(F(x_{1})-\frac{i-1}{n}\right)$$ [Eqn 10]

#### Anderson–Darling test

Anderson–Darling (*A*^2^) is a goodness-of-fit test in which the fitting of an observed continuous PDF (sample) to an expected continuous PDF (parent) is carried out.

The test statistic *A*^2^ is defined as (Mzezewa, Misi & Van Rensburg 2010):

$$A^{2}=-n-\frac{i}{n}\sum_{i=1}^{n}(2i-1)\left[ln\;F(x_{1})+\text{ln}\left(1-F(x_{n-i+1})\right)\right]$$ [Eqn 11]

where *n* is the number of events in the sample.

### Identification of candidate theoretical continuous probability distribution functions

From the AMDR data, probability–probability(P–P) plots were constructed and applied to visually identify the candidate TCPDFs for analysis of magnitude and frequency of AMDR events. The application of P–P plots involved plotting ECPDF values against TCPDF values of 50 continuous and discrete PDFs supported by EasyFit software (Mathwave 2015). The three TCPDF curves closest to the diagonal of the plots were selected as the candidate TCPDFs (Mathwave 2015).

Quantile–quantile (Q–Q) plots were applied to confirm the identified candidate TCPDFs. The application of Q–Q plot involved plotting the AMDR data: *X_i_* (i = 1 … . .55) against the X-axis, and the corresponding values against the Y- axis: *F*^−1^(*F_n_* (*X_i_*) – 0.5/n), where *F*^−1^(*X*) is the inverse cumulative TCPDF and *F_n_* (*X*) is the ECPDF. The proximity of the TCPDF plots to the displayed diagonal in EasyFit graph indicated the confirmation of the candidate TCPDFs (Mathwave 2015).

### The best-fit theoretical continuous probability distribution function

Based on the test statistics: *X*^2^, KS and *A*^2^ of each candidate TCPDF, the candidate TCPDFs were ranked. The candidate TCPDF with the lowest test statistic was ranked 1 and the candidate TCPDF with the largest test statistic was ranked 3. The total number of identified candidate TCPDF was 3. The candidate TCPDF with the least sum of ranks from the three goodness-of-fit was considered to be the best-fit TCPDF for the analysis of magnitude and frequency of the AMDR events.

### Quantile function

The parameters of the identified best-fit TCPDF were estimated using maximum likelihood method using EasyFit 5.5 software (Mathwave 2015:7). Based on the PDF of the identified best-fit TCPDF, the estimated parameters were applied to develop quantile function (QF) (Mathwave 2015). The developed QF was applied to estimate quantiles of AMDR of return periods of 2, 3, 4, 5, 10, 15, 20, 25 and 50 years.

To determine how well the developed QF fitted the AMDR events, a probability difference (P–D) plot was constructed. P–D plot is a plot of the difference between the ECPDF and the TCPDF (Mathwave 2015):

$$\text{Diff }(\text{x})=F_{n}(X)-F(X)$$ [Eqn 12]

where *F_n_* (*X*) was the ECPDF and *F* (*X*) was the identified best-fit TCPDF which was generalised logistic (GL).

### Confidence intervals of estimated quantiles

The model (equation 13) proposed by Stedinger, Vogel and Foufoula-Georgiou (1993) to estimate 95% confidence intervals of quantiles estimated by the application of GL distribution function was applied to determine the confidence intervals of estimated quantiles of return periods of 2, 3, 4, 10, 15, 20, 25 and 50 years:

(x^T−ZI−∞2{x^T},x^T+XI−∞2{x^T}) [Eqn 13]

where $Z_{I}-\frac{\infty }{2}$ is the upper 100 (∝/2)% percentile of the normal distribution. *x*^^^ *_T_* is the estimated quantile of return period (T).

## Results and discussions

### Descriptive statistics of annual maximum daily rainfall events

The descriptive statistics of AMDR events of Nelspruit for the period 1961–2015 are presented in Table 2.

**TABLE 2:** Descriptive statistics of maximum annual daily rainfall (1961–2015).

Statistic value	Sample	Range	Mean	Variance	Standard deviation	Coefficient of variation	Standard error	Skewness	Excess kurtosis
V	55	126	62.74	611.87	24.74	0.39	3.33	0.09	0.87

The mean of AMDR events was 62.74 mm. The median was 58.00 mm. The Skewness of the AMDR events was positive (0.09), indicating that the TCPDF of AMDR events had the tail that was on the right. This tail was longer or taller than on the left. These figures indicated that the AMDR events were not normally distributed.

### The plotting positions and return periods

Equation 2 expresses the relationship between plotting positions of AMDR events and the corresponding empirical return periods that are presented in Table 2. The empirical return period of 100 mm AMDR event that has been adopted in this study as a threshold is 9.17 years (Table 3). Results of several studies have also shown that the return period of flood hazards that result in flood disasters in Nelspruit and its environs is 10 years (Kelbe 1984).

**TABLE 3:** Plotting positions and return periods.

R	X (mm)	Pi	T (years)
1	126.00	0.02	55.00
2	112.60	0.04	27.50
3	110.50	0.05	18.33
4	106.20	0.07	13.75
5	102.40	0.90	11.00
6	100.30	0.11	9.17
7	89.20	0.13	7.86
8	88.40	0.15	6.88
9	83.50	0.16	6.11
10	81.40	0.18	5.50
11	79.30	0.20	5.00
12	79.00	0.22	4.58
13	78.00	0.24	4.23
14	77.00	0.25	3.93
15	75.60	0.27	3.67
16	74.80	0.29	3.44
17	73.20	0.31	3.24
18	72.40	0.33	3.06
19	72.20	0.35	2.89
20	68.80	0.36	2.75
21	67.20	0.38	2.62
22	67.00	0.40	2.50
23	65.10	0.42	2.39
24	64.70	0.44	2.29
25	64.50	0.45	2.20
26	60.50	0.47	2.12
27	59.40	0.49	2.04
28	58.00	0.51	1.96
29	57.90	0.53	1.90
30	57.70	0.55	1.83
31	57.60	0.56	1.77
32	57.06	0.58	1.72
33	57.00	0.60	1.67
34	56.50	0.62	1.62
35	56.20	0.64	1.57
36	56.00	0.65	1.53
37	55.60	0.67	1.49
38	55.30	0.69	1.45
39	52.20	0.71	1.41
40	49.50	0.73	1.38
41	48.00	0.75	1.34
42	47.40	0.76	1.31
43	46.80	0.78	1.28
44	46.00	0.80	1.25
45	44.20	0.82	1.22
46	43.50	0.84	1.20
47	40.60	0.85	1.17
48	37.80	0.87	1.15
49	37.70	0.89	1.12
50	37.10	0.91	1.10
51	36.80	0.93	1.08
52	34.60	0.95	1.06
53	24.50	0.96	1.04
54	0.00	0.98	1.02
54	0.00	0.98	1.02

### Flood disaster risk analysis results

In this study, the AMDR event of magnitude 100 mm was taken as threshold (*x*_*T*_) as indicated in sections above. AMDR events equal to or greater than *x*_*T*_ were considered as flood disaster events. Taking *x*_*T*_ = 100 mm, from the sample data in this study (Table 3), the number of occurrence of events *x*_*T*_ ≥ 100 mm was 6, and the number of intervals was 5. Therefore, the empirical return period of event *X*_*T*_ was 10 years (55/5 = 10 years).

The exceedance probability of flood disaster event (AMDR event: X ≥ 100 mm) at least once in 1, 2, 3, 4 and 5 years calculated by applying equation 6 is presented in Table 4.

**TABLE 4:** Exceedance probability of flood disaster event (X ≥ 100 mm).

Year	1	2	3	4	5
P (> 100)	0.10	0.19	0.27	0.34	0.41

The exceedance probability of flood disaster event (≥ 100 mm) occurring at least once in 5 years is 0.41. The results are important for decision-making on the acceptable level of risk because of flooding that should be associated with designing specific to infrastructure for the reduction of the risk of flood in Nelspruit and environs.

### Candidate theoretical continuous probability distribution functions

**FIGURE 3:**
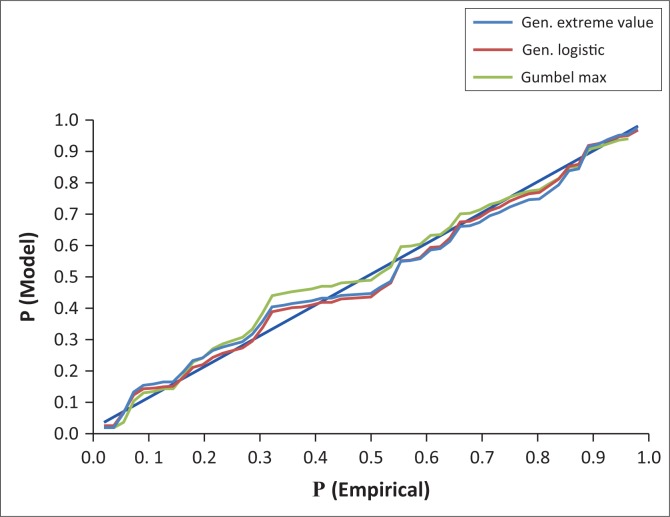
Probability–probability plot of annual maximum daily rainfall events for Nelspruit (1961–2015).

**FIGURE 4:**
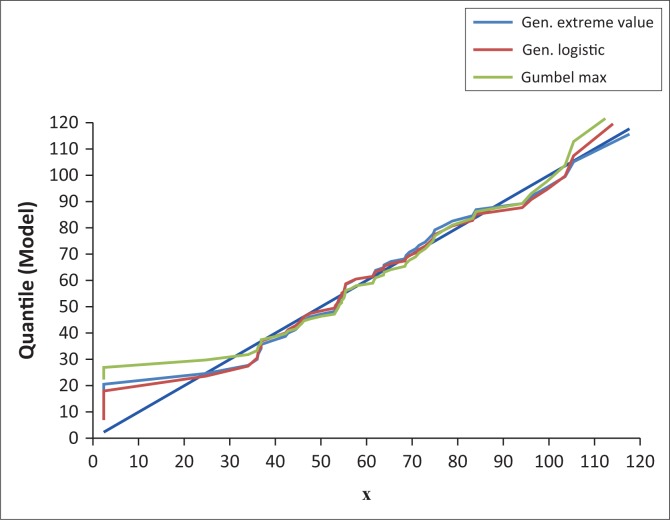
Quantile–quantile plot of annual maximum daily rainfall events for Nelspruit (1961–2015).

Applying P–P and Q–Q plots, GL, GEV and Gumbel maximum (GM) were identified as the candidate TCPDFs for frequency analysis of the AMDR events by visual identification method. The developed plots are presented in Figures 3 and 4.

The P–P plot (Figure 3) showed that GL, GEV and GM distribution functions were closest to the plot diagonal. This indicated that the GL, GEV and GM were the candidate functions for frequency analysis of the AMDR events (Mathwave 2015). However, it was not possible to identify the best-fit TCPDF for describing the AMDR events. The Q–Q plot (Figure 4) showed that GL, GEV and GM distribution functions underestimated AMDR quantiles of magnitudes < 40 mm. The three functions accurately estimated AMDR quantiles of magnitudes of the range 40 mm – 95 mm. Between 95 mm and 110 mm, all the three models overestimated the quantiles. Quantiles > 110 mm were underestimated by GL and GEV but overestimated by GM. The diagonal line of the Q–Q plot was the reference. If a distribution function curve lays on the diagonal, this indicates that the function accurately estimates the quantiles (Mathwave 2015).

### Best-fit theoretical continuous probability distribution function

The results of identifying the best-fit PDF for frequency analysis of the AMDR events for Nelspruit and environs are presented in Table 5.

**TABLE 5:** Best-fit distribution.

TCPDF	Kolmogorov–Smirnov		Anderson–Darling		Chi-squared		Rank sum
	Statistic	Rank	Statistic	Rank	Statistic	Rank	
Generalised extreme value (GEV)	0.09125	2	0.56426	2	4.6075	3	7
Generalised logistic (GL)	0.07556	1	0.31126	1	3.0771	1	3
Gumbel max (GM)	0.12881	3	1.18240	3	3.3621	2	8

TCPDF, Theoretical continuous probability distribution functions.

The rankings of the candidate TCPDFs as the best-fit TCPDFs for the frequency analysis of AMDR events based on statistics of the three goodness-of-fit tests are presented in Table 5. The candidate TCPDF with the least sum of the rankings was GL distribution. GL distribution was therefore identified as the best-fit TCPDF because it had the lowest sum of rankings.

### Generalised logistic probability distribution function

Generalised logistic PDF is defined by the QF as (Shin et al. 2011):

$$X_{T}=\sum +\frac{\alpha }{\beta }\left[1-{\left(T-1\right)}^{-\beta }\right]$$ [Eqn 14]

α is the scale parameter, *β* is the shape parameter, Σ is the location parameter.

The parameters, Σ, α and *β*, were estimated by applying the method of maximum likelihood (ML) in Mathwave software (Mathwave 2015). These parameters are shown in Table 6.

**TABLE 6:** Estimated parameters.

Distribution	Parameters
Generalised logistic	*β* = 0.05, *α* =13.58, ∑ = 61.61

The specific QF of GL probability function for estimating AMDR events for Nelspruit and environs was developed based on the parameters in Table 6 as indicated in Equation 15:

$$X_{T}=61.61+271.60\left[1-{\left(T-1\right)}^{-0.05}\right]$$ [Eqn 15]

Equation 15 was applied to estimate quantiles of return periods 2, 3, 4, 5, 10, 15, 20, 25 and 50 years. The estimated quantiles are presented in Table 7.

**TABLE 7:** Quantile–return period.

RT(YRS)	2	3	4	5	10	15	20	25	50
X_*T*_ (mm)	61.61	70.86	76.13	79.80	89.87	95.19	98.79	101.51	109.64

**FIGURE 5:**
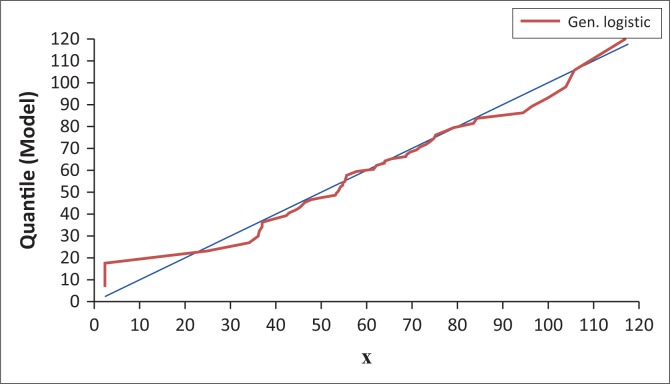
Quantile–quantile plot of the annual maximum daily rainfall events for Nelspruit (1961–2015).

The estimated quantiles of the AMDR events of return periods between 2 and 50 years which were obtained by applying equation 15 are presented in Table 7. The comparison of results of magnitude analysis of the AMDR events based on ECPDFs and TCPDFs (Table 7) shows that GL PDF fairly accurately estimates the AMDR events of magnitudes 61.61 mm – 101.51 mm. The performance of GL PDF in estimating magnitudes of AMDR events is further demonstrated by the Q–Q plot results. The Q–Q plot based on GL PDF is presented in Figure 5.

The GL PDF was identified as the best-fit TCPDF in this study (Table 5). It should be noted that GL PDF accurately estimated the annual maximum daily rainfall events of range 40 mm – 90 mm (Figure 5). This range corresponds to quantiles of return period range 1.38–7.86 years (Table 1, Figure 5). GL PDF overestimated AMDR events of range 90 mm – 112 mm (Figure 5). This range corresponds to quantiles of return period range 9.17–27.50 years (Table 1, Figure 5). GL PDF under estimated the annual maximum daily rainfall events which were > 112 mm (Figure 5). These results demonstrate the fact that even the magnitude estimates obtained from the identified best-fit TCPDF should be used in infrastructure design with caution.

### P–D plot results

**FIGURE 6:**
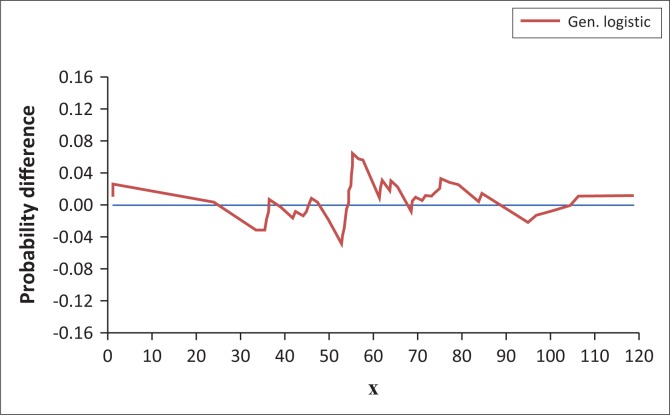
Probability–difference plot between empirical continuous probability distribution function and generalised logistic probability distribution function for annual maximum daily rainfall events for Nelspruit (1961–2015).

The results of probability difference (P–D) plot between ECPDF (based on the sample) and GL PDF are presented in Figure 6.

The P–D plot (Figure 6) showed that the probability difference between the AMDR events (the sample) and the quantiles estimated by GL > 100 mm is < 0.03 (3%). This showed that GL estimated the quantiles > 100 mm fairly accurately.

### Confidence intervals

The 95% confidence interval of the estimated quantiles is presented in Table 8.

**TABLE 8:** Confidence intervals of estimated quantiles.

Return period T (years)	2	3	4	5	10	15	20	25	50
*x*^^^ *_T_* Upper limit	67.92	82.14	93.22	96.89	122.00	135.89	138.37	143.53	154.17
*x*^^^ *_T_*	61.61	70.86	76.86	79.80	89.87	95.19	98.79	101.51	109.64
*x*^^^ *_T_* Lower limit	55.30	56.02	57.91	58.01	63.91	64.51	65.23	66.41	67.32

From the confidence intervals, the relative size of uncertainty at different return periods and the importance of different sources of uncertainty could be determined (Kjeldsen & Jones 2004). However, this was not part of this study.

## Discussions of the results

### Identification of the threshold

From the literature, AMDR events of magnitude 100 mm or greater led to flood hazards which resulted in flood disasters in Nelspruit and its environs. Records of flood disasters from 1974 to 2014 cited in this article indicated that flood hazards which resulted in flood disasters in that period were caused by AMDR events of magnitudes ≥ 100 mm. The AMDR event of magnitude 100 mm was adopted as a threshold. The AMDR events of magnitude ≥ 100 mm were assumed to be flood disaster events.

### Return period based on empirical distribution function and plotting point method

From the empirical distribution function frequency analysis, the return period of 100 mm AMDR event was 10 years. The return period of 100 mm AMDR event was 9.17 years according to plotting point applying Weibull (1939) method (Table 3). The estimate of the return periods from the two methods was in agreement. The results indicated that if the design return period of a hydraulic infrastructure being designed is less or equal to data record period, estimation of quantiles by empirical distribution function or plotting point methods is recommendable.

### Best-fit theoretical continuous probability distribution function

Generalised logistic probability distribution function was identified as the best-fit function for frequency analysis of AMDR events in Nelspruit and environs. In other studies elsewhere, different TCPDFs have been identified as the best-fit TCPDFs for frequency analysis of AMDR events. In USA, GEV was identified as the best-fit PDF for frequency analysis of AMDR events (Bonnin et al. 2006; Naghavi & Yu 1995). In Malaysia, Daud et al. (2002) identified Log-Gumbel as the best-fit function for frequency analysis of AMDR events. In Cote de Ivoire, Goula Bi et al. (2010) identified Gumbel and Lognormal functions as the best-fit TCPDFs for frequency analysis of AMDR events in Cote de Ivoire, and Smithers and Schulze (2001) proposed Log Pearson 3 function as the best-fit PDF for AMDR events in South Africa.

The performance of GL TCPDF was assessed by applying Q–Q and P–D plots (Figures 4 and 5). Both plots affirmed that the performance of GL was satisfactory. However, there seem to be no universally accepted TCPDF for frequency analysis of AMDR events. Therefore, it is advisable to carry out best-fit TCPDF identification study first for designing any hydro meteorological infrastructure projects in specific regions or sites rather than just adopting any TCPDF.

### Practical implication

The results of the study are applicable in flood hazard mapping. The results can also be applied in formulating mitigation of flood hazard in two ways. An engineering approach in designing flood control structures, for example designing and planning storm water and flood management and control infrastructure like urban drainage systems, culverts and bridges. Regulatory approach designed to reduce vulnerability, for example flood plain zoning. The results of the study can also be applied in formulating methods of flood disaster risk reduction which include, among others, among others disaster preparedness.

### Limitations

The main limitation of the study was the short period of record of daily rainfall events data from which the AMDR events were extracted. The record was only 55 years. Length of record data affects the performance of empirical distribution function to estimate return period of magnitudes and therefore the associated risks. The performance is poor with short records. Deciding on the magnitude of AMDR events as a threshold for frequency analysis was another limitation.

### Recommendations

Further studies on the topics below are recommended:

- Hydrological deterministic studies should be carried out to determine the critical magnitude of AMDR events which result in flood disaster. The magnitude of 100 mm applied in this study was adopted from literature.
- Further studies are recommended for determining the best-fit PDF for analysis of frequency and magnitude of AMDR events. The goodness-of-fit method applied in this study should be tested with data from other catchments.
- A study on the effect of climate change on magnitude and frequency of AMDR events should be carried out.

## Conclusion

The main objective of this study was to carry out statistical analysis of AMDR events for Nelspruit and its environs. From literature, AMDR events of magnitude 100 mm or more were identified as events that cause flood hazards resulting in flood disasters in Nelspruit and its environs. From the study, the return period of AMDR event of magnitude 100 mm in Nelspruit and its environs was 10 years. The occurrence probability of AMDR events of magnitude 100 mm or more at least once was 0.01, 0.19, 0.27, 0.34 and 0.41 in 1, 2, 3, 4 and 5 years, respectively. These results can be applied in developing systems that can be applied in disaster risk reduction in Nelspruit and its environs.

From the study, GL, GEV and GM theoretical PDFs were identified as the suitable candidate TCPDFs for frequency analysis of maximum annual daily rainfall events of Nelspruit and its environs. From the three candidate TCPDFs, GL was identified as the best-fit TCPDF for frequency and magnitude analysis of AMDR events in Nelspruit and environs. The performance of GL as the best-fit PDF for frequency analysis of AMDR events was evaluated by the use of Q–Q and D–P plots. The performance was found to be satisfactory. It was therefore concluded that GL PDF is suitable for frequency analysis of AMDR events for Nelspruit and its environs.
